# Supplementary material for: Characterisation of a stable laboratory co-culture of acidophilic nanoorganisms
Source: Sci Rep. 2017 Jun 12;7:3289. doi: 10.1038/s41598-017-03315-6 (PMC5468238; doi:10.1038/s41598-017-03315-6)
Supplement: Supplementary file 1 — Supplementary Information [file 41598_2017_3315_MOESM1_ESM.docx]

**Supplementary Information**

Characterisation of a stable laboratory co-culture of acidophilic nanoorganisms

Susanne Krause [1], Andreas Bremges [3,4], Philipp C. Münch [3,5], Alice C. McHardy [3] and Johannes Gescher* [1,2]

[1] Department of Applied Biology, Karlsruhe Institute of Technology (KIT), Karlsruhe, Germany

[2] Institute for Biological Interfaces, Karlsruhe Institute of Technology (KIT), Eggenstein-Leopoldshafen, Germany

[3] Computational Biology of Infection Research, Helmholtz Centre for Infection Research, Braunschweig, Germany

[4] German Center for Infection Research (DZIF), partner site Hannover-Braunschweig, Braunschweig, Germany

[5] Max von Pettenkofer-Institute of Hygiene and Medical Microbiology, Ludwig-Maximilians-University of Munich, Munich, Germany

**SI Materials and Methods**

**Quantification of cells using quantitative PCR**

The target sequences were amplified by PCR from genomic DNA of the enrichment cultures using primers see table S8. The amplified fragments contained overlapping regions as well as a *BamH*I site at the 5’ end and a *Sac*I site at the 3’ end. Using the overlaps, the fragments were combined and cloned via the added restrictions sites into plasmid pAH95 ^1^. Integration in the genome of *E. coli* DH5alphaZ1 was conducted as described before ^1^.

For standard curve design, serial dilutions of *E. coli* DH5alphaZ1 cells containing the merged 23S target sequences of the ARMAN und *Thermoplasmatales* enrichments were prepared and cells were counted in a Neubauer counting chamber (Marienfeld, Lauda-Königshofen, Germany). DNA of the dilution series as well as of enrichment-cultures was extracted using the innuSPEED soil DNA kit (Analytic Jena, Jena, Germany) with minor modifications to the manufacturer´s instructions. The starting material was 0.5 ml of each sample. The cells were spun down and washed once with PBS buffer (pH 2.5). The cell pellets were resuspended in 600 µl ELS and stored at -20°C until all samples from the timeline were collected. The following thermal lyses step was extended to 40 min. Homogenization was conducted using a Mixer Mill MM 400 (Retsch, Haan, Germany) at 30 Hz for 7 min. DNA was finally eluted in 40 µl elution buffer.

# qPCR reactions and analyzes were performed in a CFX96 Cycler (Bio-Rad, Munich, Germany). The optimal annealing temperature of 53°C was determined by a temperature gradient qPCR using isolated DNA from the developed *E. coli* strain. The qPCR reaction mix was prepared according to the manufacturer´s instructions of the SsoAdvanced™ Universal SYBR^®^ Green Supermix (Bio-Rad, Munich, Germany) with a final primer concentration of 0.5 µM and 1 µl template DNA. Conditions for qPCR were chosen as follows: initial denaturation at 95°C for 7 min, 32 cycles of 95°C for 10 sec, 53°C for 15 sec and 65°C for 30 sec, followed by a melting curve analysis of 60°C – 98°C with 0.1°C/sec. Standard curves were developed using biological triplicates, while samples from the enrichment cultures were quantified using technical duplicates of biological triplicates.

**MCL analysis**

Clusters of orthologous genes were inferred using OrthoMCL ^2^ (v. 2.0, percent-match cutoff set to 50, e-value-exponent cutoff set to -15, minimal length set to 10, max percent stop set to 20, granularity set to 1.5) as done in Hacquard et al. (2016)^3^. For annotation, HMM models were generated for each KEGG Orthologue (KO) group from the database ^4^ using the HMMER toolkit ^5^ (v. 3.1b2). We employed the HMM models to search all predicted ORFs using hmmsearch (v. 3.1b2, E-value cut-off set to 0.001) and selected the hits with best expected values for the annotation. Multiple testing correction was applied based on FDR with alpha set to 0.5.

## **Metagenome assembly and binning**

All metagenomic reads from replicates S1 and S2 were jointly assembled with Ray Meta (v2.3.1; ^6^), using a *k*-mer size of 31. Assembler and *k*-mer size were empirically selected to maximize contiguity and inclusivity (*i.e.* the percentage of included reads) of the metagenome assembly. As a proxy for contiguity, we compared N50 values, as determined by QUAST (v3.1; ^7^). To estimate the inclusivity, we aligned all metagenomic reads to the assembled contigs with Bowtie 2 (v2.2.4^8^) and calculated the mapping statistics with SAMtools (v1.1^9^).

MetaBAT (v0.23.1^10^) was used in its “very specific” mode to recover genome bins from our metagenome assembly. MetaBAT is an unsupervised binning tool that leverages nucleotide composition – in particular tetranucleotide frequencies – and per-sample differential coverage information to group contigs into genome bins. Upon manual inspection of the automatically generated four genome bins, we merged two partial genome bins into one, leading to three final genome bins. Lastly, we estimated each bin’s completeness and contamination with CheckM (v1.0.4; ^11^) and assigned a taxonomy to each contig with taxator-tk (v1.2.1; ^12^).

The genome comparisons were visualized as Circos plots^13^ using Donut (unpublished; https://github.com/hzi-bifo/Donut) on selected KEGG terms (Supplementary Table S4). We used prodigal v. 2.6.3^14^ to detect ORFs within genomes and prokka v. 1.2^15^ for their annotation. Hits to KEGG HMM were inferred with the hmmsearch command of HMMER^5^ using an E-value of 0.0001. Blastp of NCBI-blast 2.2.30^16^ with an E-value of 1e-100 was used to detect similarities of ORFs between genomes. We used a combination of samtools^9^ and bedtools v. 2.25.0^17^ to extract gene density and hypothetical gene density within a sliding window of 10000 nucleotides.

## **Functional annotation and KEGG pathway analyses**

The archaeal genome bins were annotated with Prokka (v1.11;^15^), which uses RNAmmer (v1.2^18^) and Prodigal (v2.6.0^14^) to predict ribosomal RNA genes and protein-coding genes, respectively. We then counted the number of metagenome and metatranscriptome reads within genes with BEDTools (v2.22.0^17^) and calculated their reads per kilobase (RPK) values. This information was used to determine the 23S rRNA gene copy numbers, too. The coding sequences were also annotated with the KEGG Automatic Annotation Server (v2.0^19^) to determine orthologous genes in KEGG (r78.^20^) and their corresponding KEGG pathways. For the assignments we used bi-directional best BLAST hits against 40 well-chosen organisms for covering the most common metabolic pathways, with specific focus on archaea (*neq*, *csy*, *mse*, *sai*, *iho*, *pto*, *tac*, *mac*, *msi*, *abi*, *hth*, *afo*, *pfr*, *mlu*, *mta*, *afe*, *acr*, *pde*, *gme*, *son*, *ppr*, *eco*, *aca*, *mer*, *fpl*, *nde*, *dap*, *dte*, *hya*, *rsp*, *dba*, *rfr*, *reu*, *pae*, *avn*, *acb*, *fbl*, *kcr*, *asc*, and *tvo*).

**Equipment and settings**

CARD-FISH images were taken with a Leica DM5500 B microscope using a Leica CTR5500 electronic box. The used objective was a HCX PL PLOUTAR 100×/1,30 OIL with filtercubes DAPI ET, Y3 ET and L5 ET. Images were taken with camera DFC 360 FX and software Leica LAS AF Lite.

Utilized exposure time was set to 1000 ms and 1500 ms and gain was set between 1.0 and 2.2, depending on fluorescence intensity based on grade of agglomerate formation.

# SI Figures and Tables

#
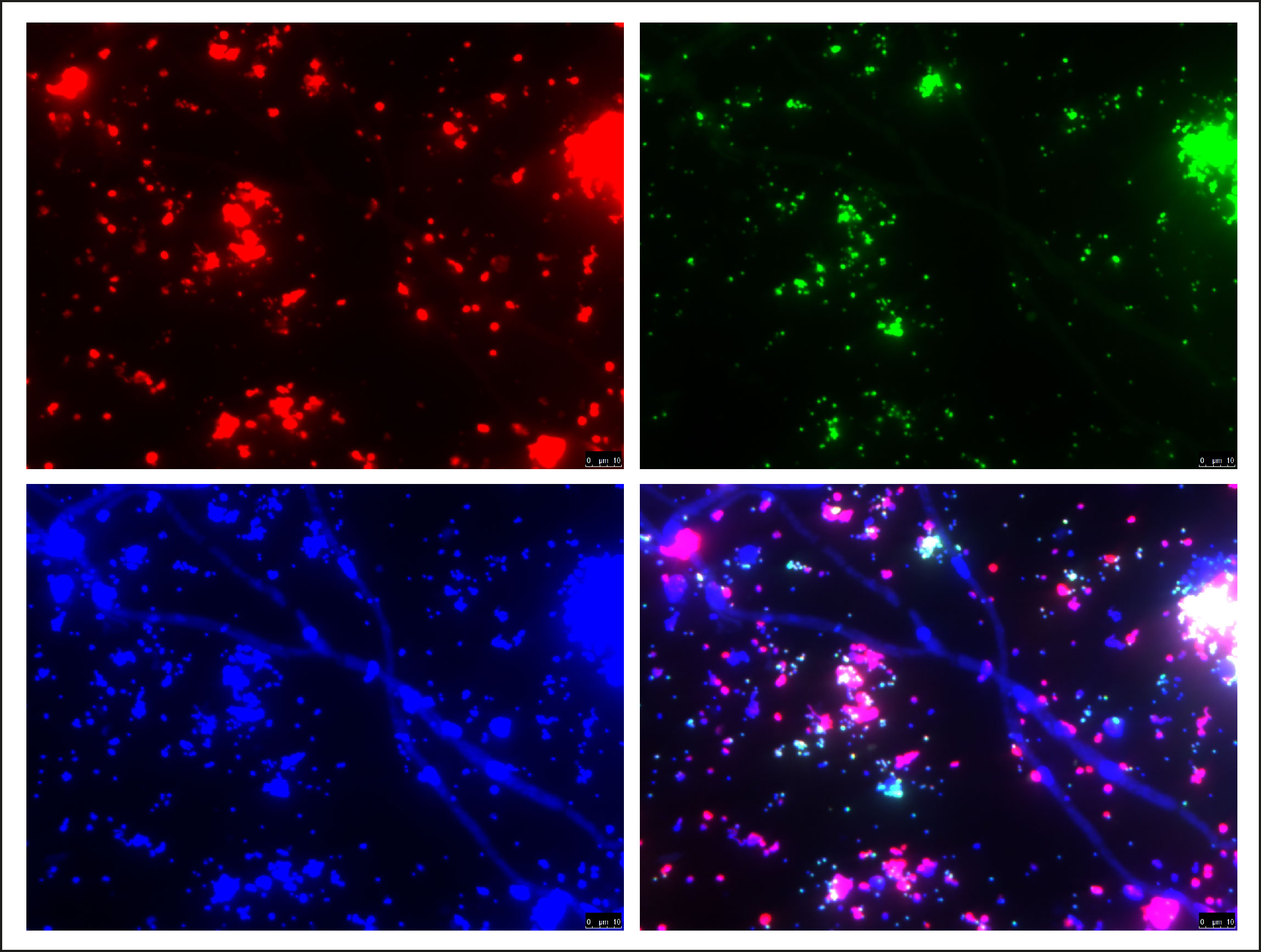


# Figure S1: CARD-FISH picture of enrichment cultures showing B_DKE and C_DKE in red (ARCH915, Alexa 546), A_DKE in green (ARM980, Alexa 488). The in blue indicated DAPI stain also displays the presence of hyphae with stained cell nuclei originating from the fungus *Acidothrix acidophila* (DAPI, blue).

**Figure S2:** Taxonomic profiles of the two metagenome replicates, S1 and S2. After taxonomic binning with Taxator-tk, we visualized community abundance profiles with Krona^21^ by weighting each contig with the number of contained reads. *Thermoplasmatales* show a high abundance, as confirmed by 16S rRNA gene sequencing, while the ARMAN-2 related sequences were reliably classified only as Archaea by the Taxator-tk software (due to a lack of publicly available reference genomes to date).


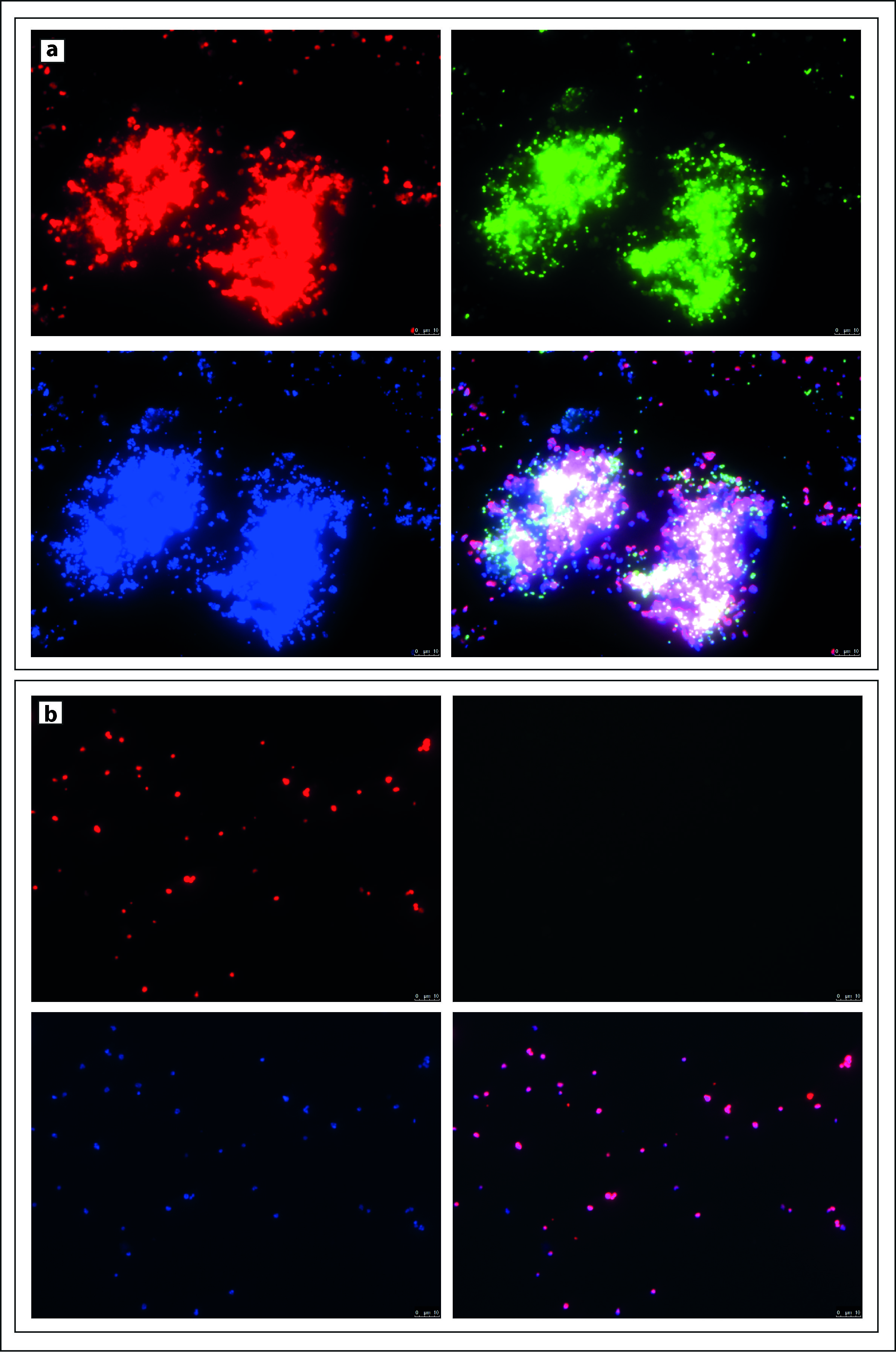


**Figure S3:** CARD-FISH pictures of enrichment cultures showing: (a) a culture containing B_DKE and C_DKE (red, ARCH915, Alexa 546) and A_DKE (green, ARM980, Alexa 488) in agglomerates and (b) an enrichment culture containing only B_DKE (red, ARCH915, Alexa 546), growing as single cells.

**Table S1:** Most closely related organisms to the 16S rRNA gene sequence of B_DKE.

| **Organism** | **Query coverage** | **E-value** | **Identity** | **Locality** | **NCBI Accession number** |
| --- | --- | --- | --- | --- | --- |
| Uncultured *Thermoplasma* sp. clone JL62 | 99% | 0.0 | 99% | Rio Tinto, Spain | HQ730609.1 |
| Uncultured *Thermoplasmatales* archaeon clone ORCL3.3 | 99% | 0.0 | 99% | Huelva, Spain | EF396244 |
| Uncultured archaeon clone 20m_arch_h3 | 99% | 0.0 | 99% | Iberian Pyritic Belt, Spain | HM745409 |
| Uncultured *Thermoplasmatales* archaeon clone S4BAC1 | 99% | 0.0 | 99% | Cae Coch, Wales | GU229859 |
| Uncultured archaeon clone AG_Eug_f6 | 99% | 0.0 | 99% | Rio Tinto, Spain | EU370310 |
| Uncultured archaeon clone CEM_Pin_c1a | 99% | 0.0 | 99% | Rio Tinto, Spain | EU370309 |
| Uncultured archaeon clone LMi-biof-arch_d6 | 99% | 0.0 | 99% | Copahue, Neuquen, Argentina | KP204537 |

**Table S2:** Metagenome assembly and binning statistics. We assembled a total of ~4.9 Mbp, of which we grouped ~4.5 Mbp (91.5%) into three near-complete genome bins. Completeness and contamination estimated based on lineage-specific marker genes.

| **Assembly** | **Assembly size [bp]** | **No. of contigs** | **N50 [bp]** | **Largest contig [bp]** | **No. of genes** | **Est. Completeness**  **[%]** | **Est. Contamination**  **[%]** |
| --- | --- | --- | --- | --- | --- | --- | --- |
| Metagenome | 4,904,630 | 145 | 178,318 | 1,202,937 | 5,272 | n/a | n/a |
| A_DKE | 976,441 | 7 | 194,415 | 336,535 | 1,013 | 81.78 | 0.93 |
| B_DKE | 1,905,249 | 31 | 82,562 | 235,491 | 2,016 | 98.61 | 0.00 |
| C_DKE | 1,606,190 | 3 | 1,202,937 | 1,202,937 | 1,556 | 86.29 | 0.00 |

**Table S3**: Functional enrichment analysis: orthoMCL groups of each genome assigned to each indicated functional category revealed significant difference between ARMAN and *Thermoplasmatales* members (two-sided Fisher's exact test, P=1.9E-8, 3.6E-8 and 5.0E-6, respectively).

| **Function** | **P-VALUE** | **in ARMAN genomes** | **in THERMO genomes** | **in ARMAN (SD)** | **in THERMO (SD)** | **FDR** | **level** |
| --- | --- | --- | --- | --- | --- | --- | --- |
| Translation | 6,70169E-10 | 88 | 182 | 3,915780041 | 1,669045921 | 1,8765E-08 | * * * |
| Amino acid metabolism | 2,57011E-09 | 106 | 852 | 12,58305739 | 16,3270153 | 3,5981E-08 | * * * |
| Infectious diseases | 5,32072E-07 | 38 | 60 | 3,109126351 | 1,8516402 | 4,9660E-06 | * * * |
| Metabolism of cofactors and vitamins | 8,29063E-07 | 86 | 671 | 5,916079783 | 13,50595107 | 5,8034E-06 | * * * |
| Carbohydrate metabolism | 5,91152E-05 | 93 | 656 | 4,272001873 | 12,17726218 | 3,3104E-04 | * * * |
| Metabolism of terpenoids and polyketides | 0,000435318 | 22 | 213 | 4,509249753 | 2,875388173 | 1,5236E-03 | * * |
| Replication and repair | 0,000354307 | 43 | 103 | 0,957427108 | 3,720119046 | 1,5236E-03 | * * |
| Transcription | 0,000406985 | 19 | 30 | 1,5 | 1,669045921 | 1,5236E-03 | * * |
| Signal transduction | 0,000722403 | 53 | 142 | 5,909032634 | 3,991061441 | 2,2475E-03 | * * |
| Folding, sorting and degradation | 0,000946476 | 57 | 159 | 1,892969449 | 3,563204817 | 2,6501E-03 | * * |
| Nucleotide metabolism | 0,004129785 | 73 | 233 | 1,5 | 4,155461123 | 1,0512E-02 | * |
| Energy metabolism | 0,005618563 | 111 | 390 | 14,97497913 | 7,667184248 | 1,3110E-02 | * |
| Cell growth and death | 0,017245843 | 23 | 58 | 2,217355783 | 3,370036032 | 3,7145E-02 | * |
| Cell motility | 0,018843852 | 5 | 5 | 2,121320344 | 0 | 3,7688E-02 | * |
| Endocrine system | 0,061058894 | 5 | 8 | 0,5 | 0 | 1,1398E-01 |  |
| Membrane transport | 0,135787818 | 116 | 631 | 4,242640687 | 16,4093832 | 2,3549E-01 |  |
| Nervous system | 0,142976928 | 0 | 12 | NA | 0 | 2,3549E-01 |  |
| Overview | 0,20672122 | 14 | 97 | 2,380476143 | 3,090885218 | 3,2157E-01 |  |
| Metabolism of other amino acids | 0,242326219 | 6 | 15 | 1,732050808 | 0,353553391 | 3,5711E-01 |  |
| Cancers | 0,261566786 | 4 | 9 | 0,577350269 | 0,487950036 | 3,6619E-01 |  |
| Xenobiotics biodegradation and metabolism | 0,650319455 | 41 | 178 | 3,593976442 | 10,1664715 | 8,6709E-01 |  |
| Lipid metabolism | 0,702545485 | 17 | 91 | 2,061552813 | 1,597989809 | 8,9415E-01 |  |
| Biosynthesis of other secondary metabolites | 0,79109035 | 16 | 83 | 1,414213562 | 1,302470181 | 9,6307E-01 |  |
| Drug resistance | 1 | 8 | 38 | 0 | 2,187627547 | 1,0000E+00 |  |
| Glycan biosynthesis and metabolism | 1 | 8 | 39 | 1,414213562 | 0,83452296 | 1,0000E+00 |  |
| Environmental adaptation | 1 | 0 | 3 | NA | 0 | 1,0000E+00 |  |
| Immune diseases | 1 | 0 | 2 | NA | 0 | 1,0000E+00 |  |
| Transport and catabolism | 1 | 0 | 2 | NA | 0 | 1,0000E+00 |  |
|  |  |  |  |  |  |  |  |
| *** for FDR corrected P value < 0.001 |  |  |  |  |  |  |  |
| ** for FDR corrected P value < 0.01 |  |  |  |  |  |  |  |
| * for FDR corrected P value < 0.05 |  |  |  |  |  |  |  |

**Table S4:** KEGG terms used for Circos plot preparation.

| **Glycolysis/**  **Gluconeogenesis** | **Citric acid cycle** | **Pentsoe phosphate pathway** | **Entner Doudoroff pathway** | **Oxydative phosphorylation** | **Beta oxidation** |
| --- | --- | --- | --- | --- | --- |
| K00134 | K00024 | K00033 | K00036 | K00239 | K00232 |
| K00150 | K00030 | K00036 | K00131 | K00240 | K00249 |
| K00844 | K00031 | K00615 | K00134 | K00241 | K00255 |
| K00845 | K00116 | K00616 | K00874 | K00242 | K06445 |
| K00850 | K00164 | K01057 | K00927 | K00244 | K01692 |
| K00873 | K00174 | K01783 | K01057 | K00245 | K13767 |
| K00886 | K00175 | K01807 | K01625 | K00246 | K07516 |
| K00918 | K00176 | K01808 | K01690 | K00247 | K01825 |
| K00927 | K00177 | K01810 | K03738 | K00330 | K01782 |
| K01086 | K00239 | K06859 | K07404 | K00331 | K00632 |
| K01596 | K00240 | K07404 |  | K00332 | K07508 |
| K01610 | K00241 | K08093 |  | K00333 |  |
| K01622 | K00242 | K08094 |  | K00334 |  |
| K01623 | K00244 | K13810 |  | K00335 |  |
| K01624 | K00245 | K13831 |  | K00336 |  |
| K01689 | K00246 | K15916 |  | K00337 |  |
| K01803 | K00247 |  |  | K00338 |  |
| K01810 | K00382 |  |  | K00339 |  |
| K01834 | K00658 |  |  | K00340 |  |
| K02446 | K01647 |  |  | K00341 |  |
| K03841 | K01676 |  |  | K00342 |  |
| K04041 | K01677 |  |  | K00343 |  |
| K06859 | K01678 |  |  | K00404 |  |
| K11389 | K01679 |  |  | K00405 |  |
| K11532 | K01681 |  |  | K00406 |  |
| K11645 | K01682 |  |  | K00407 |  |
| K13810 | K01902 |  |  | K00410 |  |
| K15633 | K01903 |  |  | K00411 |  |
| K15634 | K18118 |  |  | K00412 |  |
| K15635 |  |  |  | K00413 |  |
| K15916 |  |  |  | K00425 |  |
| K16305 |  |  |  | K00426 |  |
| K16306 |  |  |  | K02107 |  |
| K16370 |  |  |  | K02108 |  |
|  |  |  |  | K02109 |  |
|  |  |  |  | K02110 |  |
|  |  |  |  | K02111 |  |
|  |  |  |  | K02112 |  |
|  |  |  |  | K02113 |  |
|  |  |  |  | K02114 |  |
|  |  |  |  | K02115 |  |
|  |  |  |  | K02117 |  |
|  |  |  |  | K02118 |  |
|  |  |  |  | K02119 |  |
|  |  |  |  | K02120 |  |
|  |  |  |  | K02121 |  |
|  |  |  |  | K02122 |  |
|  |  |  |  | K02123 |  |
|  |  |  |  | K02124 |  |
|  |  |  |  | K02128 |  |
|  |  |  |  | K02258 |  |
|  |  |  |  | K02259 |  |
|  |  |  |  | K02274 |  |
|  |  |  |  | K02275 |  |
|  |  |  |  | K02276 |  |
|  |  |  |  | K02277 |  |
|  |  |  |  | K02297 |  |
|  |  |  |  | K02298 |  |
|  |  |  |  | K02299 |  |
|  |  |  |  | K02300 |  |
|  |  |  |  | K02826 |  |
|  |  |  |  | K02827 |  |
|  |  |  |  | K02828 |  |
|  |  |  |  | K02829 |  |
|  |  |  |  | K03886 |  |
|  |  |  |  | K03887 |  |
|  |  |  |  | K03888 |  |
|  |  |  |  | K03889 |  |
|  |  |  |  | K03890 |  |
|  |  |  |  | K03891 |  |
|  |  |  |  | K03940 |  |
|  |  |  |  | K03941 |  |
|  |  |  |  | K03943 |  |
|  |  |  |  | K05572 |  |
|  |  |  |  | K05573 |  |
|  |  |  |  | K05574 |  |
|  |  |  |  | K05575 |  |
|  |  |  |  | K05576 |  |
|  |  |  |  | K05577 |  |
|  |  |  |  | K05578 |  |
|  |  |  |  | K05579 |  |
|  |  |  |  | K05580 |  |
|  |  |  |  | K05581 |  |
|  |  |  |  | K05582 |  |
|  |  |  |  | K05583 |  |
|  |  |  |  | K05584 |  |
|  |  |  |  | K05585 |  |
|  |  |  |  | K13378 |  |
|  |  |  |  | K13380 |  |
|  |  |  |  | K15408 |  |
|  |  |  |  | K15862 |  |

**Table S5:** Annotated gene families that are unique to A_DKE compared to the ARMAN genomes used in this study.

| **ID** | **KEGG number** | **Function lvl1** | **Function lvl2** | **Function lvl3** | **Description** |
| --- | --- | --- | --- | --- | --- |
| gfam_377 | K13832 | Metabolism | Amino acid metabolism | Phenylalanin, tyrosine and tryptophan biosynthesis | aroDE, DHQ-SDH, 3-dehydroquinate dehydratase / shikimate dehydrogenase [EC:4.2.1.10 1.1.1.25] |
| gfam_405 | K01597 | Metabolism | Metabolism of terpenoids and polyketides | Terpenoid backbone biosynthesis | MVD, mvaD, diphosphomevalonate decarboxylase [EC:4.1.1.33] |
| gfam_414 | K12454 | Metabolism | Carbohydrate metabolism | Amino sugar and nucleotide sugar metabolism | rfbE, CDP-paratose 2-epimerase [EC:5.1.3.10] |
| gfam_423 | K16160 | Metabolism | Energy metabolism | Methane metabolism | mmoB, methane monooxygenase regulatory protein B |
| gfam_426 | K01597 | Metabolism | Metabolism of terpenoids and polyketides | Terpenoid backbone biosynthesis | MVD, mvaD, diphosphomevalonate decarboxylase [EC:4.1.1.33] |
| gfam_436 | K13950 | Metabolism | Metabolism of cofactors and vitamins | Folate biosynthesis | pabAB, para-aminobenzoate synthetase [EC:2.6.1.85] |
| gfam_552 | K03524 | Metabolism | Metabolism of cofactors and vitamins | Biotin metabolism | birA, BirA family transcriptional regulator, biotin operon repressor / biotin-[acetyl-CoA-carboxylase] ligase [EC:6.3.4.15] |
| gfam_1082 | K16150 | Metabolism | Carbohydrate metabolism | Starch and sucrose metabolism | K16150, glycogen(starch) synthase [EC:2.4.1.11] |
| gfam_1128 | K11434 | Environmental Information Processing | Signal transduction | FoxO signaling pathway | PRMT1, protein arginine N-methyltransferase 1 [EC:2.1.1.-] |
| gfam_1741 | K18232 | Environmental Information Processing | Membrane transport | ABC transporters | oleC4, oleandomycin transport system ATP-binding protein |
| gfam_1753 | K00857 | Metabolism | Nucleotide metabolism | Pyrimidine metabolism | tdk, TK, thymidine kinase [EC:2.7.1.21] |
| gfam_1907 | K10216 | Metabolism | Overview | Degradation of aromatic compounds | dmpD, xylF, 2-hydroxymuconate-semialdehyde hydrolase [EC:3.7.1.9] |
| gfam_2934 | K16785 | Environmental Information Processing | Membrane transport | ABC transporters | ecfT, energy-coupling factor transport system permease protein |
| gfam_3121 | K01241 | Metabolism | Nucleotide metabolism | Purine metabolism | amn, AMP nucleosidase [EC:3.2.2.4] |

**Table S9:** Primers used in this study.

| **Name** | **Sequence 5´ to 3´** | **Application** |
| --- | --- | --- |
| ITS1 | TCCGTAGGTGAACCTGCGG | General forward primer for 5,8S rRNA gene and relating ITS DNA-sequences of fungi ^22^ |
| ITS4 | TCCTCCGCTTATTGATATGC | General reverse primer for 5,8S rRNA gene and relating ITS DNA-sequences of fungi ^22^ |
| Fusionprimer for | GTGTTTTACGCCGCATCC | Forward fusion primer for A_DKE and *Thermoplasmatales* sequences (this study) |
| Fusionprimer rev | CAGTGATAAGCTGTCAAACATGAG | Reverse fusion primer for A_DKE and *Thermoplasmatales* sequences (this study) |
| A_DKE cloning Primer for | GTGTTTTACGCCGCATCCGGCATTACAAAATGACTTTGTAAACGCGTGGATCCCTTAGTACAGTGGGTGACGC | Forward cloning primer for A_DKE (this study) |
| A_DKE cloning Primer rev | CAGACGACATCGAAGTAGCAAACCCTATGGTTGGGACAGTGGAACAATC | Reverse cloning primer for A_DKE (this study) |
| *Thermoplasmatale*s cloning Primer for | TGGCGTAACGATTGTTCCACTGTCCCAACCATAGGGTTTGCTACTTCGA | Forward cloning primer for *Thermoplasmatales* (this study) |
| *Thermoplasmatale*s cloning Primer rev | CAGTGATAAGCTGTCAAACATGAGAATTCGAGCTCCGTTCCTCTCGTACTAAAGGC | Reverse cloning primer for *Thermoplasmatales* (this study) |
| A_DKE qPCR for | CTTAGTACAGTGGGTGACGC | Forward qPCR primer for A_DKE (this study) |
| A_DKE qPCR rev | GGTTGGGACAGTGGAACAATC | Reverse qPCR primer for A_DKE (this study) |
| Thermo qPCR for | GGGTTTGCTACTTCGATGTCG | Forward qPCR primer for *Thermoplasmatales* (this study) |
| Thermo qPCR rev | CGTTCCTCTCGTACTAAAGGC | Forward qPCR primer for *Thermoplasmatales* (this study) |
| Arch20F | TTCCGGTTGATCCYGCCRG | General forward primer for 16S rRNA gene of archaea^23^ |
| Uni1406R | GACGGGCRGTGTGTRCAA | General forward primer for 16S rRNA gene of prokaryotes^24^ |

**Table S10:** Calculating 23S rRNA gene copy numbers. Normalized per-sample coverages for the 23S rRNA genes are below the average/median metagenomic coverages of all genes, indicating a 23S rRNA gene copy number of one, respectively. RPK = Reads per kilobase.

|  | **RPK in S1** | | | **RPK in S2** | | |
| --- | --- | --- | --- | --- | --- | --- |
| **Genome** | **23S rRNA gene** | **Median of all genes** | **Mean ± SD of all genes** | **23S rRNA gene** | **Median of all genes** | **Mean ± SD of all genes** |
| **A_DKE** | 75,582.5 | 96,092.1 | 96,042.1 ± 12,216.3 | 148,337.0 | 203,944.0 | 201,755.0 ± 22,531.3 |
| **B_DKE** | 113,425.0 | 141,111.0 | 141,842.0 ± 20,907.6 | 44,626.6 | 58,081.1 | 58,767.9 ± 8,550.3 |
| **C_DKE** | 18,981.8 | 19,835.6 | 19,633.0 ± 2,595.7 | 2,474.4 | 2,767.1 | 2,750.8 ± 350.1 |

# Table S11: Metagenomic and -transcriptomic sequencing. We sequenced 26.6 Gbp and 17.3 Gbp of the metagenome and –transcriptome, respectively, with biological replicates available for metagenomic samples (aiding the successive binning step).

| **Sample** | **Library** | **Protocol** | **No. of reads** | **No. of base pairs [bp]** | **Included in assembly [%]** |
| --- | --- | --- | --- | --- | --- |
| S1 | MG | 2 x 51 bp | 293,819,146 | 14,984,776,446 | 95.38 |
| S2 | MG | 2 x 51 bp | 227,249,154 | 11,589,706,854 | 96.17 |
| MT | MT | 2 x 100 bp | 173,164,004 | 17,316,400,400 | 91.83 |

**SI References**

1. Haldimann, A. & Wanner, B. L. Conditional-replication , integration , excision , and retrieval plasmid-host systems for gene structure-function studies of bacteria. *J. Bacteriol.* **183,** 6384–6393 (2001).

2. Li, L., Stoeckert, C. J. J. & Roos, D. S. OrthoMCL: Identification of Ortholog Groups for Eukaryotic Genomes. *Genome Res.* **13,** 2178–2189 (2003).

3. Hacquard, S. *et al.* Survival trade-offs in plant roots during colonization by closely related beneficial and pathogenic fungi. *Nat. Commun.* **7,** 11362 (2016).

4. Ogata, H. *et al.* KEGG: Kyoto Encyclopedia of Genes and Genomes. *Nucleic Acids Res.* **27,** 29–34 (1999).

5. Eddy, S. Profile hidden Markov models. *Bioinformatics* **14,** 755–763 (1998).

6. Boisvert, S., Raymond, F., Godzaridis, E., Laviolette, F. & Corbeil, J. Ray Meta: scalable *de novo* metagenome assembly and profiling. *Genome Biol.* **13,** R122 (2012).

7. Gurevich, A., Saveliev, V., Vyahhi, N. & Tesler, G. QUAST: Quality assessment tool for genome assemblies. *Bioinformatics* **29,** 1072–1075 (2013).

8. Langmead, B. & Salzberg, S. L. Fast gapped-read alignment with Bowtie 2. *Natl. Institutes Heal.* **9,** 357–359 (2013).

9. Li, H. *et al.* The Sequence Alignment/Map format and SAMtools. *Bioinformatics* **25,** 2078–2079 (2009).

10. Kang, D. D., Froula, J., Egan, R. & Wang, Z. MetaBAT, an efficient tool for accurately reconstructing single genomes from complex microbial communities. *PeerJ* **3,** (2015).

11. Parks, D. H., Imelfort, M., Skennerton, C. T., Hugenholtz, P. & Tyson, G. W. CheckM: assessing the quality of microbial genomes recovered from isolates, single cells, and metagenomes. *Genome Res.* **25,** 1043–55 (2015).

12. Droege, J., Gregor, I. & McHardy, A. C. Taxator-tk: Precise taxonomic assignment of metagenomes by fast approximation of evolutionary neighborhoods. *Bioinformatics* **31,** 817–824 (2015).

13. Krzywinski, M. *et al.* Circos: an Information Aesthetic for Comparative Genomics. *Genome Res* **19,** 1639–1645 (2009).

14. Hyatt, D. *et al.* Prodigal: prokaryotic gene recognition and translation initiation site identification. *BMC Bioinformatics* **11,** 119 (2010).

15. Seemann, T. Prokka: Rapid prokaryotic genome annotation. *Bioinformatics* **30,** 2068–2069 (2014).

16. Altschul, S. F., Gish, W., Miller, W., Myers, E. W. & Lipman, D. J. Basic Local Alignment Search Tool. *J. Mol. Biol.* 403–410 (1990).

17. Quinlan, A. R. & Hall, I. M. BEDTools: A flexible suite of utilities for comparing genomic features. *Bioinformatics* **26,** 841–842 (2010).

18. Lagesen, K. *et al.* RNAmmer: Consistent and rapid annotation of ribosomal RNA genes. *Nucleic Acids Res.* **35,** 3100–3108 (2007).

19. Moriya, Y., Itoh, M., Okuda, S., Yoshizawa, A. C. & Kanehisa, M. KAAS: An automatic genome annotation and pathway reconstruction server. *Nucleic Acids Res.* **35,** 182–185 (2007).

20. Kanehisa, M. & Goto, S. KEGG: Kyoto encyclopedia of genes and genomes. *Nucleic Acids Res.* **28,** 27–30 (2000).

21. Ondov, B. D., Bergman, N. H. & Phillippy, A. M. Interactive metagenomic visualization in a Web browser. *BMC Bioinformatics* **12,** 385 (2011).

22. White, T. J., Bruns, T., Lee, S. & Taylor, J. in *PCR protocols: a guide to methods and applications.* (eds. Innis, M. A., Gelfand, D. H., Sninsky, J. & White, T. J.) 315–322 (Academic Press Inc, 1990).

23. Massana, R. & Murray, A. Vertical distribution and phylogenetic characterization of marine planktonic Archaea in the Santa Barbara Channel. *Appl. Environ. Microbiol.* **63,** 50–56 (1997).

24. Kato, S. *et al.* Abundance of Zetaproteobacteria within crustal fluids in back-arc hydrothermal fields of the Southern Mariana Trough. *Environ. Microbiol.* **11,** 3210–3222 (2009).

# SI Titles

**Table S6:** Gene families of all three archaeal community members of the enrichment culture and related organisms based on OrthoMCL analysis.

**Table S7:** KEGG transcript annotations of A_DKE, B_DKE and C_DKE of enzymes involved in central metabolism.

**Table S8:** Most abundant top 20 mRNA reads of all three community members of the enrichment culture, using KEGG and PROKKA databases for annotation.
